# Supplementary material for: Comparison of the Phenotypic Performance, Molecular Diversity, and Proteomics in Transgenic Rice
Source: Plants (Basel). 2022 Dec 29;12(1):156. doi: 10.3390/plants12010156 (PMC9824520; doi:10.3390/plants12010156)
Supplement: Supplementary file 1 [file plants-12-00156-s001.zip › plants-2035990-supplementary.pdf]

Supporting Information

Table S1 Rate(%) of deadhearts and white spikelets in transgenic lines and control line across field experiments

Tissue	Stem at tillering stage	Stem at heading stage
	Rate(%) of deadhearts	Rate(%) of white spikelets
CH891	31.45±0.87	29.31±0.05
CH891(1C) vs CH891	2.93±0.24	1.45±0.03
CH891(2A) vs CH891	2.33±0.25	1.41±0.03
CH891(1C+2A) vs CH891	1.03±0.45	0.72±0.02

Note: The data presented are the mean ± standard deviation, n = 10.

Table S2 Analysis of LSD table for CH891(1C), CH891(2A), CH891(1C+2A) and CH891 across field experiments

Trait	Group	Mean value	standard deviation	Letter label LSD
Panicle length	CH891	24.61	2.73	a
Panicle length	CH891(1C)	26.73	1.92	b
Panicle length	CH891(2A)	23.94	3.01	a
Panicle length	CH891(1C+2A)	23.08	2.34	a
panicles per plant	CH891	7.00	0.77	a
panicles per plant	CH891(1C)	7.90	1.14	a
panicles per plant	CH891(2A)	7.40	0.92	a
panicles per plant	CH891(1C+2A)	7.00	1.18	a
grains per panicle	CH891	171.37	46.15	a
grains per panicle	CH891(1C)	162.21	29.63	a
grains per panicle	CH891(2A)	149.00	34.72	a
grains per panicle	CH891(1C+2A)	159.53	31.21	a
seed-set rate	CH891	85.73	6.34	a
seed-set rate	CH891(1C)	84.93	12.40	a
seed-set rate	CH891(2A)	82.87	8.08	a
seed-set rate	CH891(1C+2A)	89.25	8.62	b
weight per 1000-grain	CH891	23.23	0.51	a
weight per 1000-grain	CH891(1C)	23.36	0.66	a
weight per 1000-grain	CH891(2A)	23.13	0.53	a
weight per 1000-grain	CH891(1C+2A)	23.45	0.58	a
yield per plant	CH891	25.89	6.21	a
yield per plant	CH891(1C)	25.43	7.70	a
yield per plant	CH891(2A)	25.17	5.62	a
yield per plant	CH891(1C+2A)	25.16	4.76	a
brown rice rate	CH891	78.54	0.49	a
brown rice rate	CH891(1C)	74.55	0.42	c
brown rice rate	CH891(2A)	78.72	0.28	a
brown rice rate	CH891(1C+2A)	76.48	0.19	b
head rice rate	CH891	62.54	1.46	a
head rice rate	CH891(1C)	48.32	0.72	b
head rice rate	CH891(2A)	63.26	0.77	a
head rice rate	CH891(1C+2A)	62.25	0.74	a
grain length to width ratio	CH891	3.13	0.06	a
grain length to width ratio	CH891(1C)	3.16	0.04	a
grain length to width ratio	CH891(2A)	3.30	0.03	b
grain length to width ratio	CH891(1C+2A)	3.12	0.03	a
chalkiness degree	CH891	3.60	0.31	a
chalkiness degree	CH891(1C)	3.29	0.19	b
chalkiness degree	CH891(2A)	3.58	0.21	a

chalkiness degree	CH891(1C+2A)	3.35	0.59	a
gel consistency	CH891	50.20	1.35	a
gel consistency	CH891(1C)	51.15	1.08	a
gel consistency	CH891(2A)	51.20	1.34	a
gel consistency	CH891(1C+2A)	50.71	1.73	a
amylose content	CH891	15.21	1.33	a
amylose content	CH891(1C)	15.63	1.47	a
amylose content	CH891(2A)	17.07	1.22	b
amylose content	CH891(1C+2A)	13.50	1.22	b

Table S3 Relative expression level of *CRY1C* and *CRY2A* in stems of three transgenic lines at different stages

Tissue	Relative expression level of *CRY1C*/*CRY2A* ($2^{-\Delta\Delta CT}$)			
	CH891(1C)	CH891(2A)	CH891(1C+2A)	
	CRY1C	*CRY2A*	*CRY1C*	*CRY2A*
Stem at tillering stage	2.97±0.10	2.97±0.12	2.47±0.02	2.56±0.04
Stem at heading stage	15.47±0.21	13.06±0.11	5.72±0.08	12.04±0.11

Note: The data presented are the mean ± standard deviation, n = 3.

Table S4 Cry1C and Cry2A protein content (μg·g^{-1}) in stems of three transgenic lines at different stages

Tissue	Cry1C/Cry2A protein content (μg·g−1)			
	CH891(1C)	CH891(2A)	CH891(1C+2A)	
	Cry1C	Cry2A	Cry1C	Cry2A
Stem at tillering stage	1.42±0.08	11.36±0.88	0.89±0.09	10.84±0.29
Stem at heading stage	2.87±0.28	12.64±1.43	1.78±0.10	17.68±0.89

Note: The data presented are the mean ± standard deviation, n = 3.

Table S5 Physical location of molecular markers

Chr	Chromosome length/Mb	Marker_num	Average length between markers/Mb
chr1	435.10	53	8.21
chr2	358.50	53	6.76
chr3	363.00	52	6.98
chr4	351.80	45	7.82
chr5	296.70	39	7.61
chr6	309.80	40	7.75
chr7	295.60	43	6.87
chr8	280.70	43	6.53
chr9	229.20	31	7.39
chr10	227.50	37	6.15
chr11	284.00	37	7.68
chr12	273.00	39	7.00

Table S6 Genetic background response rate statistics

Lines	Number of SSR marker	Number of polymorphic SSR marker	Polymorphic marker rate / %	Theoretical genetic background response rate / %	Actual genetic background response rate / %
CH891(1C)	512	22	4.29	93. 75	97.85
CH891(1C)	512	15	2.93	93. 75	98.54
CH891(1C+2A)	512	19	3.71	93. 75	98.14
CH891	512	0	0	100	100

Table S7 Result information of COG analysis between three transgenic lines and control line

COG category	Category description	Number of proteins	Between groups of enrichment
Metabolism	secondary metabolites biosynthesis transport and catabolism	10	CH891(1C) vs CH891
Metabolism	secondary metabolites biosynthesis transport and catabolism	14	CH891(2A) vs CH891
Metabolism	secondary metabolites biosynthesis transport and catabolism	20	CH891(1C+2A) vs CH891
Metabolism	Carbohydrate transport and metabolism	18	CH891(1C) vs CH891
Metabolism	Carbohydrate transport and metabolism	25	CH891(2A) vs CH891
Metabolism	Carbohydrate transport and metabolism	20	CH891(1C+2A) vs CH891
Cellular processes and signaling	Posttranslational modification, protein turnover, chaperones	33	CH891(1C) vs CH891
Cellular processes and signaling	Posttranslational modification, protein turnover, chaperones	41	CH891(2A) vs CH891
Cellular processes and signaling	Posttranslational modification, protein turnover, chaperones	44	CH891(1C+2A) vs CH891
Poorly characterize	Function unknown	22	CH891(1C) vs CH891
Poorly characterize	Function unknown	30	CH891(2A) vs CH891
Poorly characterize	Function unknown	27	CH891(1C+2A) vs CH891
Cellular processes and signaling	Signal transduction mechanisms	9	CH891(1C) vs CH891
Cellular processes and signaling	Signal transduction mechanisms	29	CH891(2A) vs CH891
Cellular processes and signaling	Signal transduction mechanisms	25	CH891(1C+2A) vs CH891
Information storage and processing	Translation ribosomal structure and biogenesis	13	CH891(1C) vs CH891
Information storage and processing	Translation ribosomal structure and biogenesis	15	CH891(2A) vs CH891
Information storage and processing	Translation ribosomal structure and biogenesis	14	CH891(1C+2A) vs CH891
Metabolism	Energy production and conversion	22	CH891(1C) vs CH891
Metabolism	Energy production and conversion	15	CH891(2A) vs CH891
Metabolism	Energy production and conversion	14	CH891(1C+2A) vs CH891
Metabolism	Lipid transport and metabolism	12	CH891(1C) vs CH891
Metabolism	Lipid transport and metabolism	11	CH891(2A) vs CH891
Metabolism	Lipid transport and metabolism	11	CH891(1C+2A) vs CH891
Metabolism	Amino acid transport and metabolism	13	CH891(1C) vs CH891
Metabolism	Amino acid transport and metabolism	14	CH891(2A) vs CH891
Metabolism	Amino acid transport and metabolism	11	CH891(1C+2A) vs CH891
Metabolism	Inorganic ion transport and metabolism	7	CH891(1C) vs CH891
Metabolism	Inorganic ion transport and metabolism	13	CH891(2A) vs CH891
Metabolism	Inorganic ion transport and metabolism	10	CH891(1C+2A) vs CH891
Metabolism	Coenzyme transport and metabolism	3	CH891(1C) vs CH891
Metabolism	Coenzyme transport and metabolism	6	CH891(2A) vs CH891
Metabolism	Coenzyme transport and metabolism	6	CH891(1C+2A) vs CH891
Metabolism	Nucleotide transport and metabolism	3	CH891(1C) vs CH891
Metabolism	Nucleotide transport and metabolism	5	CH891(2A) vs CH891

Metabolism	Nucleotide transport and metabolism	4	CH891(1C+2A) vs CH891
Information storage and processing	RNA processing and modification	15	CH891(1C) vs CH891
Information storage and processing	RNA processing and modification	11	CH891(2A) vs CH891
Information storage and processing	RNA processing and modification	14	CH891(1C+2A) vs CH891
Information storage and processing	Replication, recombination and repair	6	CH891(1C) vs CH891
Information storage and processing	Replication, recombination and repair	12	CH891(2A) vs CH891
Information storage and processing	Replication, recombination and repair	8	CH891(1C+2A) vs CH891
Information storage and processing	Transcription	7	CH891(1C) vs CH891
Information storage and processing	Transcription	14	CH891(2A) vs CH891
Information storage and processing	Transcription	8	CH891(1C+2A) vs CH891
Information storage and processing	Chromatin structure and dynamics	2	CH891(1C) vs CH891
Information storage and processing	Chromatin structure and dynamics	5	CH891(2A) vs CH891
Information storage and processing	Chromatin structure and dynamics	4	CH891(1C+2A) vs CH891
Cellular processes and signaling	Intracellular trafficking, secretion, and vesicular transport	7	CH891(1C) vs CH891
Cellular processes and signaling	Intracellular trafficking, secretion, and vesicular transport	30	CH891(2A) vs CH891
Cellular processes and signaling	Intracellular trafficking, secretion, and vesicular transport	15	CH891(1C+2A) vs CH891
Cellular processes and signaling	Defense mechanisms	3	CH891(1C) vs CH891
Cellular processes and signaling	Defense mechanisms	7	CH891(2A) vs CH891
Cellular processes and signaling	Defense mechanisms	8	CH891(1C+2A) vs CH891
Cellular processes and signaling	Cytoskeleton	3	CH891(1C) vs CH891
Cellular processes and signaling	Cytoskeleton	7	CH891(2A) vs CH891
Cellular processes and signaling	Cytoskeleton	7	CH891(1C+2A) vs CH891
Cellular processes and signaling	Cell cycle control, cell division, chromosome partitioning	4	CH891(1C) vs CH891
Cellular processes and signaling	Cell cycle control, cell division, chromosome partitioning	6	CH891(2A) vs CH891
Cellular processes and signaling	Cell cycle control, cell division, chromosome partitioning	7	CH891(1C+2A) vs CH891
Cellular processes and signaling	Cell wall/membrane/envelope biogenesis	2	CH891(1C) vs CH891
Cellular processes and signaling	Cell wall/membrane/envelope biogenesis	3	CH891(2A) vs CH891
Cellular processes and signaling	Cell wall/membrane/envelope biogenesis	4	CH891(1C+2A) vs CH891
Cellular processes and signaling	Nuclear structure	0	CH891(1C) vs CH891
Cellular processes and signaling	Nuclear structure	1	CH891(2A) vs CH891
Cellular processes and signaling	Nuclear structure	2	CH891(1C+2A) vs CH891

Table S8 Result information of KEGG pathway enrichment between three transgenic lines and control line

Terms	KEGG pathway name	Mapping	Fold enrichment	-log10(p value)	Between groups of enrichment
map00400	Phenylalanine, tyrosine and tryptophan biosynthesis	5	2.84	1.54	CH891(1C) vs CH891
map00400	Phenylalanine, tyrosine and tryptophan biosynthesis	6	3.02	1.90	CH891(2A) vs CH891
map00400	Phenylalanine, tyrosine and tryptophan biosynthesis	5	2.51	1.34	CH891(1C+2A) vs CH891
map00260	Glycine, serine and threonine metabolism	7	2.77	1.92	CH891(1C) vs CH891
map00260	Glycine, serine and threonine metabolism	7	2.20	1.43	CH891(2A) vs CH891
map00260	Glycine, serine and threonine metabolism	6	5.49	3.20	CH891(1C+2A) vs CH891

Table S9 PCR primers used in this study

Primer	Primer sequence	Description
CRY1C-F	AAAGAATCGCTGAGTTCGCTAG	qRT-PCR
CRY1C-R	AAGAAGTCCATCAAGGATACGG	
CRY2A-F	TTCCTGCTGAAATAAGGTGGGT	qRT-PCR
CRY2A-R	ACGAGCGAGGGTGTCAGTGTT	
Actin-F	TTCGGACCCAAGAATGCTAAG	qRT-PCR
Actin-R	AACAGATAGGCCGGTTGAAAAC	
N1-CRY1C-F	TTTCGCCATGTGTTGAGCATA	Nester PCR 1
N1-*CRY1C*-R	TGGAATTGTGAGCGGATAACA	
N2-*CRY2A*-F	CCAGTACTAAAATCCAGATCCCC	Nester PCR 2
N2-*CRY2A*-R	TCACACAGGAAACAGCTATGACC	

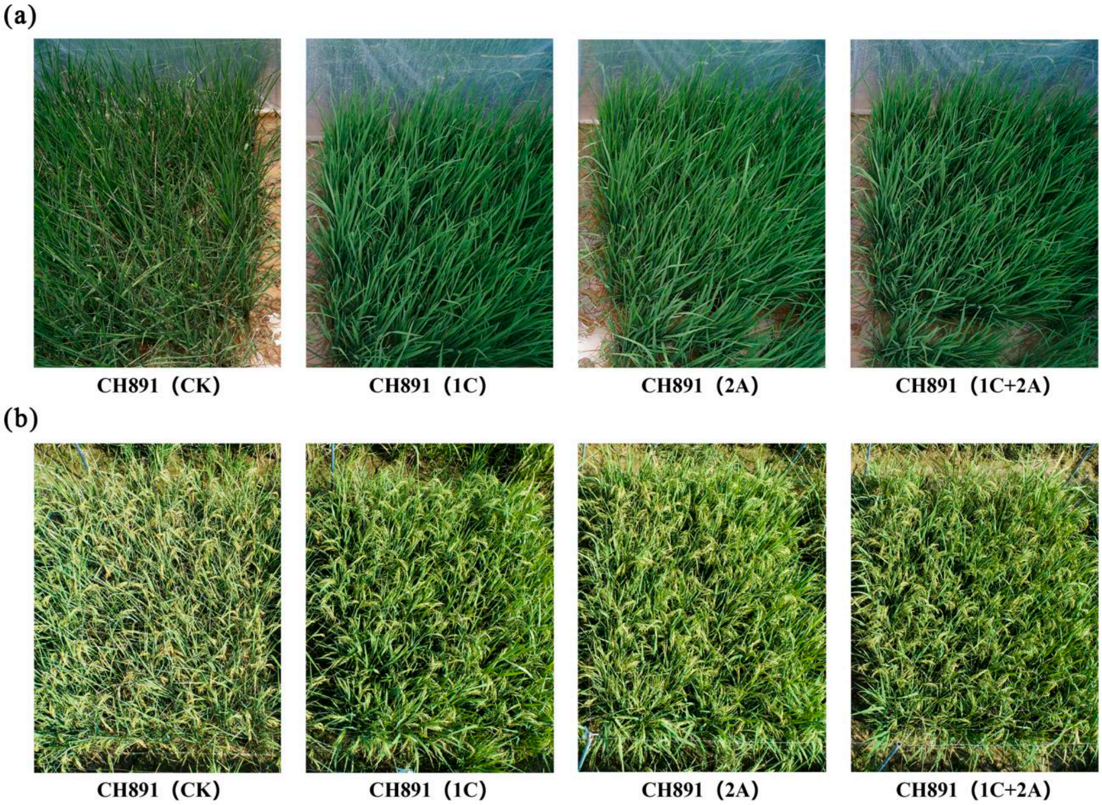

Figure S1. Field performance for insect resistance of CH891 modified CH891(1C), CH891(2A), and CH891(1C+2A) rice lines grown in Nanchang field (E115.5°, N28.5°), China. **a**. Field phenotype of transgenic restorer lines and recurrent parents exposed naturally to the insect at the tillering stage. **b**. Field phenotype of transgenic restorer lines and recurrent parents exposed naturally to the insect at the heading stage.

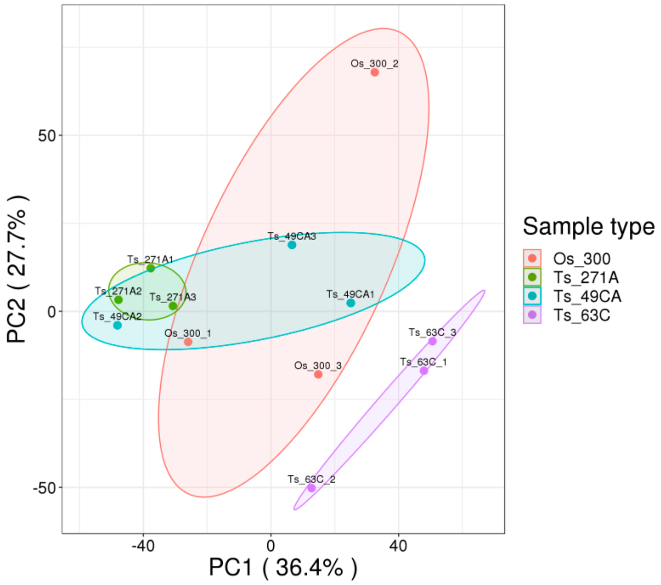

Figure S2. Principal components (PCs) analyses of protein expression levels in leaves of 4 rice lines.

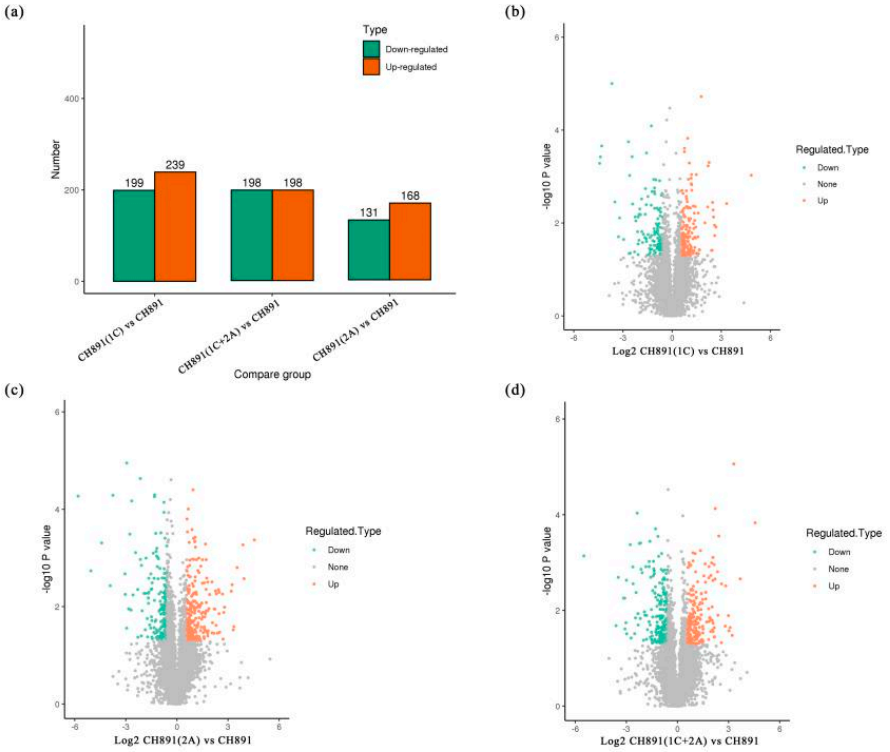

Figure S3. Statistical comparison of differentially expressed proteins. **a** Statistics of differentially expressed proteins (DEPs) among samples of NIL groups. **b** Volcano plot of DEPs between CH891(1C) and CH891. **c** Volcano plot of DEPs between CH891(2A) and CH891. **d** Volcano plot of DEPs between CH891(1C+2A) and CH891. The ordinate represents the multiple of difference and the ordinate represents the level of significance. The scatter in the upper left corner represents significant protein expression, differentially upregulated. The scatter in the upper right represents significant gene/protein expression, differentially upregulated.

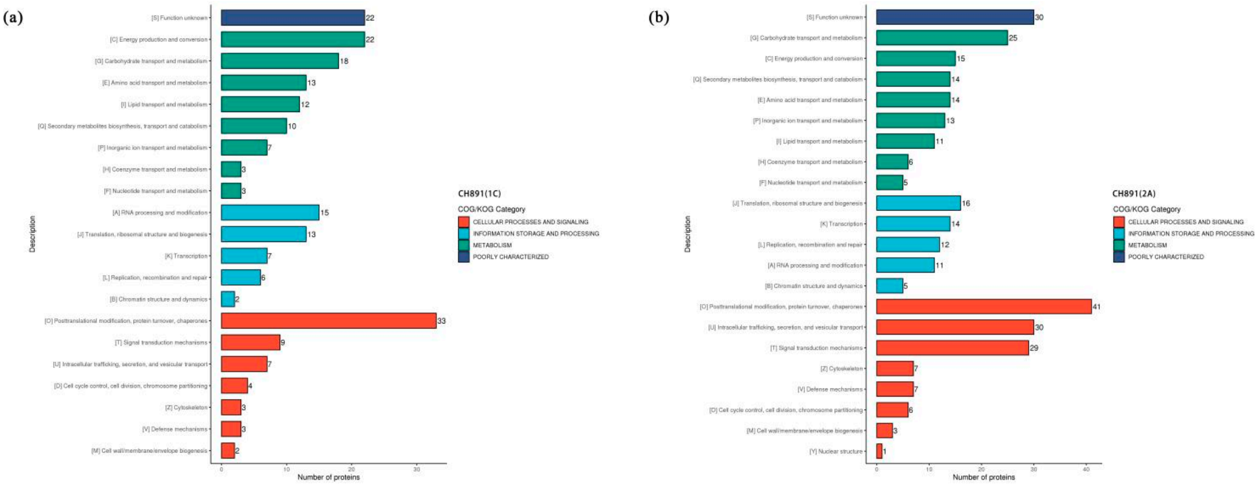

Figure S4. Cluster analysis of differentially expressed proteins (DEPs). **a** Clusters of Orthologous Groups of proteins (COG) displayed the degree of accumulation of conserved proteins among samples of NIL groups CH891(1C). **b** Clusters of Orthologous Groups of proteins (COG) displayed the degree of accumulation of conserved proteins among samples of NIL groups CH891(2A).
